# Adipose Tissue Dysfunction Related to Climate Change and Air Pollution: Understanding the Metabolic Consequences

**DOI:** 10.3390/ijms25147849

**Published:** 2024-07-18

**Authors:** Radoslav Stojchevski, Preethi Chandrasekaran, Nikola Hadzi-Petrushev, Mitko Mladenov, Dimiter Avtanski

**Affiliations:** 1 Friedman Diabetes Institute, Lenox Hill Hospital, Northwell Health, New York, NY 10003, USA; rstojchevski@northwell.edu; 2 Feinstein Institutes for Medical Research, Manhasset, NY 11030, USA; 3 Donald and Barbara Zucker School of Medicine at Hofstra/Northwell, Hempstead, NY 11549, USA; 4 UT Southwestern Medical Center Dallas, Dallas, TX 75390, USA; preethi.chandrasekaran@utsouthwestern.edu; 5 Faculty of Natural Sciences and Mathematics, Institute of Biology, Ss. Cyril and Methodius University, 1000 Skopje, North Macedonia; nikola@pmf.ukim.mk (N.H.-P.); mitkom@pmf.ukim.mk (M.M.)

**Keywords:** climate change, adiposity, WAT, BAT, air pollution, obesity, adipose tissue dysfunctions

## Abstract

Obesity, a global pandemic, poses a major threat to healthcare systems worldwide. Adipose tissue, the energy-storing organ during excessive energy intake, functions as a thermoregulator, interacting with other tissues to regulate systemic metabolism. Specifically, brown adipose tissue (BAT) is positively associated with an increased resistance to obesity, due to its thermogenic function in the presence of uncoupled protein 1 (UCP1). Recently, studies on climate change and the influence of environmental pollutants on energy homeostasis and obesity have drawn increasing attention. The reciprocal relationship between increasing adiposity and increasing temperatures results in reduced adaptive thermogenesis, decreased physical activity, and increased carbon footprint production. In addition, the impact of climate change makes obese individuals more prone to developing type 2 diabetes mellitus (T2DM). An impaired response to heat stress, compromised vasodilation, and sweating increase the risk of diabetes-related comorbidities. This comprehensive review provides information about the effects of climate change on obesity and adipose tissue, the risk of T2DM development, and insights into the environmental pollutants causing adipose tissue dysfunction and obesity. The effects of altered dietary patterns on adiposity and adaptation strategies to mitigate the detrimental effects of climate change are also discussed.

## 1. Introduction

Obesity, the condition of excessive fat accumulation, occurs when energy intake is greater than energy expenditure, resulting in an energy surplus stored in white adipose tissue (WAT). This imbalance in energy homeostasis leads to various metabolic disorders, including type 2 diabetes mellitus (T2DM). On the other hand, brown adipose tissue (BAT), characterized by brown multilocular adipocytes, stimulates thermogenesis, increasing energy expenditure to combat excessive fat accumulation in WAT, thereby emerging as a promising target for treating obesity and metabolic disorders [1,2]. Obesity is a significant risk factor for the development and progression of T2DM [3]. The prevalence of T2DM is further amplified by unhealthy dietary patterns, obesity, and physical inactivity [4]. As global health challenges, both obesity and T2DM are influenced by various environmental factors, including climate change and rising air pollution levels.

The current literature supports the hypothesis that some environmental pollutants, such as dichlorodiphenyltrichloroethane (DDT) and its metabolite dichlordiphenylethylene (DDE), are associated with increased obesity by impairing the mass and function of BAT. DDE and DDT are persistent organic pollutants that were widely used as pesticides in the past and continue to be present in the environment due to their slow degradation [5]. Air pollutants, particularly fine particulate matter (PM2.5), induce insulin resistance (IR) due to BAT mitochondrial dysfunction [6]. BAT is stimulated by cold exposure and insulin and is inversely correlated with body mass index (BMI). In addition, alterations in thermogenic gene expression are key features of obesity and IR [2].

The link between increasing adiposity and rising temperatures leads to reduced adaptive thermogenesis, decreased physical activity, and increased carbon footprint production. Additionally, the impact of climate change makes obese individuals more prone to developing T2DM. Impaired responses to heat stress, compromised vasodilation, and sweating due to the effects of climate change increase the risk of diabetes-related comorbidities.

This review aims to provide a comprehensive analysis of the effects of climate change, air pollution, and environmental factors on adipose tissue function and metabolic health. We also explore how rising temperatures and environmental pollutants affect WAT and BAT, contributing to obesity and T2DM. Additionally, we discuss potential adaptation and mitigation strategies with which to address the adverse effects of these global challenges.

## 2. Adipose Tissue and Metabolic Health

Adipose tissue functions as a metabolic sink, playing a versatile role in regulating lipid metabolism and glucose homeostasis. Metabolic diseases, such as IR, inflammation, lipid overload, and endoplasmic reticulum (ER) stress, are closely linked to adipose tissue dysfunction. Dysfunctional adipose tissue leads to differences in adipocyte characteristics and the distribution of fat deposits in obese individuals [7]. Under surplus energy conditions, adipocytes synthesize triglycerides (TGs) from the free fatty acids (FFAs) released into circulation, in addition to utilizing the fatty acids converted from acetyl CoA within the cells by de novo lipogenesis [8]. Additionally, the size of the adipocytes increases (hypertrophy), and additional adipocytes are recruited from the pre-adipocytes (hyperplasia). During these processes, the extensive tissue remodeling and activation of inflammation that occurs subsequently lead to obesity, IR, and metabolic dysfunction [8]. In line with this, adverse metabolic consequences, such as the accumulation of visceral fat in ectopic sites, dyslipidemia, and lipodystrophy, are evident [9,10].

It is important to understand the role of adipose tissue in glucose homeostasis. Thermogenic adipose tissue serves as a glucose sink under adrenergic stimulation, and the expression of glucose transporter type 4 (GLUT4) participates in peripheral glucose disposal [11]. The key hormones released by adipose tissue include leptin, adiponectin, and resistin. Leptin increases energy expenditure, and its levels are correlated with adipose tissue mass. Obese states are characterized by leptin resistance, and consequently, increased leptin levels act as a compensatory mechanism [12]. On the other hand, adiponectin suppresses hepatic glucose production and enhances muscle glucose uptake [13]. 

Recent studies have further elucidated the mechanisms linking adipose tissue dysfunction to metabolic disorders and obesity, highlighting the roles of impaired adipogenesis, altered adipokine secretion, chronic low-grade inflammation, increased FFA levels, and ectopic lipid accumulation [14,15]. The balance between lipogenesis and lipolysis is disturbed in obesity due to adipose tissue inflammation and increased tumor necrosis factor alpha (TNFα) levels, which interfere with insulin signaling [10,16]. The ectopic lipid accumulation in insulin-responsive metabolic tissues (also known as lipotoxicity) impairs insulin signaling [4].

### 2.1. WAT and Metabolic Health

WAT is the main type of body fat, categorized into two key subgroups: subcutaneous WAT (sWAT), located under the skin, and visceral WAT (vWAT), found around the abdominal organs [7]. sWAT is a main depot for lipid storage [17]. It provides insulation, protection against infections, and mechanical stress relief [8]. vWAT is usually present in small amounts in healthy individuals and is highly metabolically active, releasing FFAs into the bloodstream. In obesity, excess fat accumulates in the vWAT and other ectopic sites, such as around the heart, blood vessels, digestive organs, liver, and kidneys. This leads to insulin overproduction and resistance, inflammation, and fat deposits in the arteries [8,9].

WAT is the primary site for energy storage, in the form of triacylglycerols, and exhibits high plasticity. Thus, WAT has the ability to expand, reduce, and remodel in response to various metabolic stimuli, such as diet, exercise, and obesity [18]. The ability of sWAT expansion is the key determinant of metabolic dysregulation in obesity [19]. When there is an energy imbalance, the physiological capacity of WAT to accommodate the excess fat is exceeded, triggering organelle stress, tissue hypoxia, the accumulation of extracellular matrix components, tissue infiltration by immune cells, mitochondrial dysfunction, and lipid droplet abnormalities [20,21,22]. Moreover, WAT functions as an important endocrine organ by secreting various endocrine factors, such as adipokines, hormones, growth, and inflammatory, which regulate metabolic processes, inflammation, and insulin sensitivity. These secretions play crucial roles in maintaining energy balance and overall metabolic health.

### 2.2. BAT and Metabolic Health

BAT’s uniqueness lies in its expression of uncoupled protein 1 (UCP1), an inner mitochondrial membrane protein responsible for thermogenesis by uncoupling the mitochondrial proton gradient from ATP production to generate heat [17]. Another class of adipocytes, known as beige adipocytes, expresses UCP1 but utilizes UCP1-independent thermogenic mechanisms, such as Ca^2+^ cycling [17].

In addition to the regulation of thermogenesis, BAT is involved in crosstalk with several peripheral tissues, such as the liver, skeletal muscle, and immune cells, to regulate systemic energy balance and glucose homeostasis [23]. It is interesting to note that BAT secretes BATokines, such as fibroblast growth factor 21 (FGF21), interleukin-6 (IL-6), growth differentiation factor 15 (GDF-15), and others [24]. Studies have revealed that human pluripotent cells derived from brown adipocytes significantly improve glucose and lipid metabolism and prevent obesity [25]. Recent studies have reported the association of human BAT with lower TG levels, blood glucose, and higher high-density lipoprotein (HDL) levels [26]. In response to acute or mild cold exposure, BAT activation maintains the thermal demands through non-shivering thermogenesis [27]. Cold acclimation increases the oxidative capacity of BAT, which correlates with a reduction in shivering thermogenesis. In addition, cold adaptation in BAT is also associated with mitochondrial remodeling and vascularization for adaptive thermogenesis and fatty oxidation through UCP1 during periods of high metabolic demands [28,29]. The physiological regulation of BAT is mediated mainly via beta-3-adrenergic receptors present in brown adipocytes [30]. Additionally, BAT has a critical role in glucose metabolism. The translocation of GLUT1 and GLUT4 to the plasma membrane of brown adipocytes is induced by the stimulation of adrenergic signaling by cold exposure [11]. The uptake of glucose by BAT is also regulated by insulin signaling via the phosphatidylinositol 3-kinase (PI3K)–phosphoinositide-dependent kinase-1 (PDK1)–protein kinase B (PKB/Akt) signaling pathway, promoting the translocation of GLUT4 to the plasma membrane [11].

The major strategy for treating obesity and metabolic disorders is the manipulation of WAT to the human-like phenotype with increased thermogenic capacity, through a process called “adipocyte browning” [31].

## 3. Effects of Climate Change on Adipose Tissue

Climate change significantly impacts adipose tissue function and metabolism, exacerbating the prevalence of obesity and metabolic disorders. The rising temperatures associated with global warming impair BAT thermogenesis, reducing energy expenditure and increasing adiposity [32]. Furthermore, climate change-related factors, such as air pollution and altered dietary patterns, disrupt adipose tissue homeostasis, increasing the risk of metabolic dysfunction [33]. The rise in temperatures also challenges thermoregulation in endothermic species, placing a burden on compensatory mechanisms and raising the risk of heat stress [34]. These environmental stressors, along with extreme weather events and deforestation, further aggravate the issue by influencing nutrition, physical activity levels, and overall metabolic health [35].

The role of adipose tissue in maintaining energy homeostasis is essential to the pathophysiology of metabolic disorders. Several reactions, such as vasoconstriction and piloerection, are known to maintain the core body temperature in mammals in response to thermal challenges [36]. Shivering thermogenesis is an acute response to thermal stress, presenting as a continual contraction and relaxation of muscles. In contrast, non-shivering thermogenesis occurs in BAT, generating heat during chronic cold exposure, which is a long-term strategy to respond to cold challenges [37,38].

**Figure 1:**
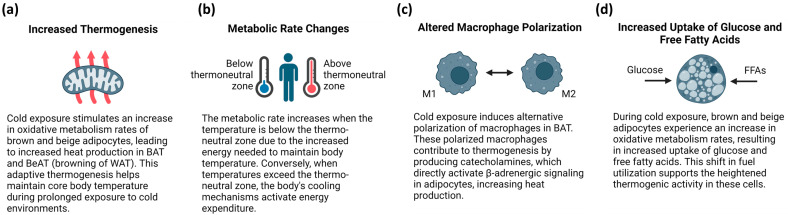
Adipose tissue responses to thermal challenges. (**a**) In BAT, polarized macrophages, due to cold exposure, directly activate beta-adrenergic signaling, thereby increasing heat production. (**b**) Energy expenditure is triggered by the body’s cooling mechanisms when the temperature exceeds the thermoneutral zone. (**c**) The elevated thermogenic activity of brown and beige adipocytes due to cold exposure is a result of increased glucose and free fatty acid uptake. (**d**) The browning of WAT is an important mechanism in which cold exposure triggers an increase in the oxidative metabolic rates of brown and beige adipocytes. This is essential for maintaining core body temperature during prolonged cold exposure. Created with BioRender.com.

It is important to note that the metabolic rate increases when the temperature is below the thermoneutral zone, due to the increased energy required to maintain body temperature (Figure 1a). However, when the temperature exceeds the thermoneutral zone, the body’s cooling mechanisms activate energy expenditure [39] (Figure 1b). In addition to BAT, sWAT and inguinal WAT (iWAT) undergo morphological changes at different temperatures. For example, in cold environments, sWAT and iWAT undergo a browning process [40,41]. Exposure to cold temperatures induces altered polarization of the macrophages in BAT. These polarized macrophages contribute to thermogenesis by producing catecholamines that directly activate β-adrenergic signaling in adipocytes [42] (Figure 1c). Briefly, cold exposure stimulates an increase in the oxidative metabolism rates of brown and beige adipocytes, resulting in an increased uptake of glucose and free fatty acids (Figure 1d).

### 3.1. Climate Change and Obesity

It is alarming that obesity affects more than 890 million adults (or one in eight people) globally [43]. Notably, 61% of diabetic patients are obese. The global obesity rate has nearly tripled since 1975 [44]. Global warming is caused by increased greenhouse gas (GHG) emissions, such as CO_2_, methane, nitrous oxide, ozone, and fluorinated gases such as chlorofluorocarbons (CFCs) and hydrofluorocarbons (HFCs) [45]. The National Longitudinal Study of Adolescent to Adult Health (Add Health) demonstrated that the atmospheric temperature correlates with a slight increase in weight [46].

**Figure 2:**
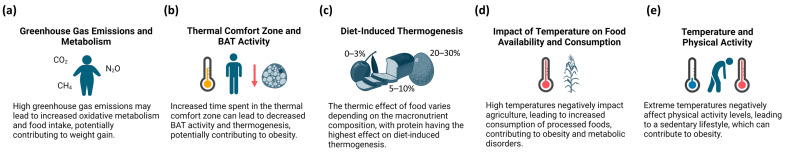
Addressing global challenges related to obesity and climate. (**a**) Obesity is triggered by high levels of greenhouse gas emissions due to increased food intake. (**b**) An important factor contributing to obesity is the increased time spent in the thermoneutral zone and decreased thermogenesis as a consequence. (**c**) A protein-rich diet is recommended due to it having the highest effect on diet-induced thermogenesis. (**d**) The increase in the consumption of processed foods is attributed to increased temperatures negatively impacting crop yields and agriculture. (**e**) Physical inactivity, as a result of extreme temperatures, contributes to weight gain. Created with BioRender.com.

The increase in oxidative metabolism due to greater metabolic demands and increased food intake may result from high GHG emissions [47] (Figure 2a). Obesity stems from many factors, such as high-calorie intake, physical inactivity, and decreased energy dissipation. Impaired thermogenesis is promoted by the reduced expression of the thermogenic genes encoding uncoupling proteins, thyroid hormone receptors, and β-adrenergic receptors, due to chemicals that disrupt hormone metabolism, which increase body weight [48].

Several studies have found that thermogenesis, a process in which brown or beige adipocytes contribute to increased energy expenditure, varies among different populations [49,50,51], and some of these variations may be attributed to the environment [52,53]. Increased time spent in the thermal neutral zone can lead to a loss of BAT and decreased thermogenic activity [54] (Figure 2b). It is established that thermogenesis plateaus above moderate physical activity levels. Regarding diet-induced thermogenesis, the energy released in the form of heat varies depending on the macronutrient composition of the food consumed. The thermic effect is lowest for fat (0–3%), followed by that for carbohydrates (5–10%), and is highest for protein (20–30%) [55,56] (Figure 2c). The variability in diet-induced thermogenesis can be attributed to factors such as sex, age, body composition, and hormonal status [57]. Exposure to ambient temperature plays an important role in BAT activity. The increased time spent in the thermal comfort zone decreases energy expenditure and has potential obesogenic consequences. At high temperatures, the neuroendocrine mechanism reduces food intake and metabolism, leading to decreased thyroid activity and testosterone and cortisol levels [58].

In contrast, low temperatures increase adrenal steroid hormone levels and the activity of the pituitary and thyroid glands. A rise in temperature negatively impacts agriculture, resulting in scarce fresh produce. Increased GHG emissions indirectly contribute to the higher production of processed foods due to multiple factors, such as the reduced availability and increased price of fresh food. The increased consumption of processed foods, characterized by their high levels of salt, sugar, and fat, leads to various health issues, including obesity and metabolic disorders [33] (Figure 2d). In addition, extreme temperatures negatively affect the level of physical activity, leading to a sedentary lifestyle [33] (Figure 2e).

### 3.2. Climate Change and T2DM

**Figure 3:**
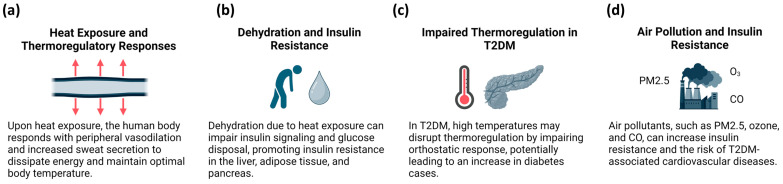
Effects of heat exposure and air pollution on insulin resistance and diabetes complications. (**a**) Exposure to high temperatures increases sweat secretion and peripheral vasodilation. This, in turn, dissipates heat and maintains optimal body temperature. (**b**) Impairment of insulin signaling stimulates insulin resistance in various tissues due to dehydration caused by heat exposure. (**c**) Glucose intolerance is a consequence of disruption of thermoregulation by impairment of orthostatic response. (**d**) Air pollutants, specifically PM2.5, increase risk of glucose intolerance and type 2 DM-associated cardiovascular diseases. Created with BioRender.com.

Upon exposure to heat, the human body responds with peripheral vasodilation, increased sweat secretion to dissipate energy, and the redistribution of blood flow to the skin. These responses cause heat loss, aiming to maintain optimal body temperature [59] (Figure 3a). Elevated blood flow to the skin may result in dehydration and the impairment of insulin signaling and glucose disposal via the inhibition of cellular insulin action and a decrease in blood flow to insulin-sensitive tissues. Furthermore, dehydration promotes IR by disrupting downstream signaling pathways, such as PI3K and hyperosmotic inhibition of PKB activation. Strikingly, increased vasopressin levels due to dehydration stimulate glucose production in the liver and promote IR in the liver, adipose tissue, and pancreas [60] (Figure 3b). In T2DM, high temperatures may also disrupt thermoregulation by impairing the orthostatic response [32]. Blauw et al. [61] estimated that each degree of C increase in the outdoor temperature may be associated with 100,000 new diabetes cases annually in the United States (Figure 3c).

Several studies have reported that air pollution increases IR and its associated complications [62,63,64,65]. Air pollutants, such as ozone and fine particulate matter (PM), can cause diabetic complications [66]. Fine particulate matter up to 2.5 μm (PM2.5) is a mixture of organic and inorganic chemicals generated from human and natural sources. It consists of carbonaceous nuclei that absorb polycyclic aromatic hydrocarbons and endotoxic metals from the atmosphere [67,68]. PM2.5 is known to increase the risk of T2DM and its associated cardiovascular diseases (CVD) [69] (Figure 3d).

## 4. Environmental Factors Affecting Adipose Tissue Metabolism

Adipose tissue undergoes hyperplasia and hypertrophy in response to energy overload and temperature changes. Perinatal exposure to endocrine disruptors, such as DDT, may impair BAT thermogenesis and increase the risk of metabolic syndrome [70]. In addition, air pollutants increase the risk of IR due to BAT mitochondrial dysfunction. The mechanism linking thermogenesis to the risk of IR involves the activation of peroxisome proliferator-activated receptor-gamma co-activator-1-alpha (PGC-1α), a master regulator of energy metabolism [70]. Furthermore, the effects of DDT and DDE on BAT may be mediated by the aryl hydrocarbon receptor (AHR), a physiological carbon regulator of energy metabolism. AHR activation is increased by pro-inflammatory cytokines [71]. In short, DDT and its metabolite DDE induce nuclear factor-kappa B (NF-κB) activation and the production of pro-inflammatory cytokines, which mediate the upregulation of the AHR [71].

### 4.1. Air Pollutants and BAT

Long-term exposure to PM2.5 has been shown to induce inflammation and decrease BAT weight, mitochondrial size in BAT, and mitochondrial number in WAT, changes associated with a process known as BAT “whitening” [68]. Interestingly, homeobox protein C9 (HOXC9) and insulin-like growth factor binding protein 3 (IGFBP3) genes, characteristic of WAT, are upregulated in BAT, supporting the transformation of brown adipocytes to the WAT phenotype [72]. Zhang et al. [6] suggested that PM2.5 might impact BAT development through TNFα-mediated apoptosis and inflammation. BAT inflammation is associated with impaired insulin signaling, as evidenced by the decreased Ser437 phosphorylation of AKT in BAT [68]. Additionally, long-term exposure to PM2.5 induces low-grade inflammation in the hypothalamus, indirectly causing BAT dysfunction. Other pollutants, such as mono-2-ethylhexyl phthalate (MEHP), promote adipocyte differentiation and induce obesity in mice [73]. A study by Farrugia et al. [74] suggested a correlation between bisphenol A (BPA) and obesity, diabetes, and metabolic disorders. In contrast, polyfluoroalkyl substances (PFAS), such as perfluorooctanoic acid (PFOA) and perfluorooctane sulfonate (PFOS), have anti-obesogenic effects, increasing the rate of oxidative capacity in brown fat mitochondria via UCP1 upregulation [75].

DDT and DDE impair BAT activity through multiple mechanisms, including reducing substrate transport and utilization, downregulating the expression of the genes involved in thermogenesis, inhibiting the deiodination of thyroxine (T4) to triiodothyronine (T3), and inducing IR and inflammatory pathways in BAT [70]. In contrast, PFOA and PFOS increase mitochondrial oxidation via UCP1 upregulation in BAT, thereby decreasing food intake and body weight.

### 4.2. Temperature-Related Adaptations of BAT Function and Metabolism

Changes in temperature alter the physiological and molecular aspects of adipose tissue to adjust to a new tissue homeostasis. Studies on mice have revealed that differences in metabolic rates have been observed due to thermal challenges. A gradual decrease in temperature from 30 °C to mild cold temperature (16–20 °C) to severe cold (5 °C) temperature causes a gradual increase in oxygen consumption [76]. Thus, when the temperature is decreased, the rate of metabolism increases as more energy is required to maintain body temperature. On the other hand, energy expenditure is stimulated when the ambient temperature exceeds the thermoneutral zone and body-cooling mechanisms are activated [77].

One study demonstrated the effects that housing *ob*/*ob* mice at 14 °C, 22 °C, and 30 °C had on their core temperature and energy expenditure. In this case, the hypothermic phenotype of the *ob*/*ob* mice was partially rescued by leptin administration associated with decreased thermal conductance, proving the physiological effects of leptin in maintaining core body temperature under sub-thermoneutral conditions [78]. The ambient temperature plays a crucial role in defining the metabolic phenotypes of mice. For example, nude mice exhibit reduced heat insulation and might activate compensatory thermogenic programs, such as BAT and beige adipocyte-mediated non-shivering thermogenesis (NST), leading to increased energy expenditure [79]. It is well known that BAT activity improves obesity-induced metabolic dysfunction. However, a lack of brown adipocytes increases body weight, IR, and adipose tissue inflammation [80]. BAT has multilocular brown adipocytes at room temperature, whereas in the thermoneutral (TN) zone, it has unilocular brown adipocytes. In addition, at cold temperatures, iWAT consists of multilocular adipocytes, indicating a browning event, which completely disappears in the TN zone [40]. Furthermore, in the TN zone, whitened BAT exhibits decreased mitochondrial density and gene and protein expression [81].

Exposure to cold induces the alternative polarization of the macrophages in BAT and beige adipose tissue (BeAT), which induces thermogenesis by producing catecholamines and directly activating β-adrenergic signaling in adipocytes [82]. Another important feature is the alteration of immune cell composition; being in the TN zone systemically causes an accumulation of lymphocyte antigen 6 complex, locus G (LYG6) + monocytes in bone marrow. Additionally, there is an increase in the TNFα and IL-6 levels in the serum of mice [83]. Conversely, cold exposure results in fewer activated monocytes and reduced T cell expression in autoimmunity [82].

Intermittent cold exposure (ICE) (exposing the body to low temperatures for short periods) is known to increase subcutaneous WAT and has variable effects on visceral WAT. ICE promotes weight loss maintenance and attenuates the positive energy balance during relapse by increasing energy expenditure in mice [84]. Numerous studies have demonstrated that ICE increases BAT activation and reduces weight [85]. ICE induces systemic responses to defend the core body temperature. For example, increased glucagon due to ICE acts as a browning stimulus via the activation of FGF21 secretion. The high expression of UCP1, high rates of substrate turnover, and abundant mitochondria are other alterations in the network of crosstalk underlying the physiological responses to ICE [86].

In BAT, the induction of UCP1 and peroxisome proliferator-activated receptor-gamma (PPARγ) expression increases the fat utilization capacity via the increased expression of lipoprotein lipase (LPL) [87]. Furthermore, beige adipocytes, which are highly responsive to cold, have increased sensitivity to irisin secreted by muscles [88]. It is essential to understand the effects of cold exposure on the secretory function of adipose tissue, particularly the modulatory role of adipokines in blood glucose and insulin sensitivity; however, direct evidence linking ICE and adipokine modulation is limited. Wang et al. [89] reported that the combination of ICE and exercise in rats reduced IR and blood glucose levels. In addition, adipose triglyceride lipase (ATGL) and LPL activity in inguinal adipose tissue were shown to increase in response to ICE. Moreover, ICE enhances the capacity of skeletal muscles to oxidize FFAs via PGC1α and p38 MAPK upregulation [89]. As much of the research is centered on rodent models, further research is needed on the effects of ICE on humans.

### 4.3. Air Pollutants and WAT Dysfunction

Chronic exposure to PM2.5 is associated with WAT expansion and increased adiposity [90]. In addition to stimulating adipogenesis, PM2.5 also decreases catecholamine-induced lipolysis. Additionally, PM2.5 exposure is associated with altered thyroid function and decreased T3 and T4 plasma levels [67,91]. In skeletal muscles, PM2.5 exposure inhibits NO-dependent microvessel dilation and decreases mitochondrial oxidative capacity [67]. Results from multiple rodent studies have suggested that exposure to PM2.5 induces adipocyte hypertrophy and WAT expansion. Notably, PM2.5 directly oxidizes organic molecules and stimulates reactive oxygen species (ROS) production, interfering with the mitochondrial respiratory chain in cells [68]. The evidence suggests that long-term exposure to PM2.5 in rodents increases the expression of lipogenic genes, such as those encoding acetyl-CoA carboxylase (ACC) and diacylglycerol O-acyltransferase 2 (DGAT2), with an increase in PPARα and cAMP response element-binding protein alpha (CREB-α) [92,93].

Importantly, exposure to PM2.5 leads to hypothalamic inflammation associated with leptin resistance, decreased energy expenditure, and WAT accumulation [94]. Additionally, it is associated with increased gut permeability, causing the migration of bacterial LPS and the release of pro-inflammatory molecules, stimulating WAT inflammation and adipogenesis. Moreover, chronic PM2.5 exposure causes a significant reduction in the mitochondrial number and size in WAT and BAT, suppressing PCG-1α and UCP1, and leading to impaired lipid metabolism, increased oxidative stress, mitochondrial dysfunction, and TG storage in white adipocytes [95,96].

### 4.4. Association between Climate Change, Air Pollution, and Altered Dietary Patterns

Unhealthy dietary habits, such as a high intake of fried and sugar-rich foods and a decreased consumption of red meat, fruits, and vegetables, contribute to central and global adiposity. Furthermore, these dietary habits are associated with sedentary behavior in adults [97]. A high intake of white bread is also associated with central and global adiposity in adults [98]. Studies have shown that food availability, access, and utilization can largely influence dietary patterns and lead to the consumption of high-calorie or processed foods. This also leads to an inadequate consumption of essential nutrients, such as proteins, vitamins, and minerals, contributing to dyslipidemia and increased central adiposity [99,100]. Climate change also affects soil fertility, rain patterns, crop yields, food production, and nutrient bioavailability [101]. It is important to note that there is a reciprocal and cyclical association between food production and climate change. Increased fertilizer use and deforestation lead to increased GHG emissions and climate change, subsequently decreasing food production [102]. Weather events, such as drought, flooding, and heat waves, correlate with decreased rain patterns, reduced soil fertility, and acid rain due to increased fertilizer usage [103]. Additionally, climate change alters supply chains, transportation, yield, biomass food composition, the quality of nutrition, and food prices. All these effects collectively increase the consumption of processed foods and high-calorie diets, thus elevating the incidence of abdominal adiposity [104]. 

Air pollution can also influence dietary patterns by affecting food production, quality, and consumption. Exposure to air pollutants, such as PM2.5 and nitrogen oxides (NO_x_), can reduce crop yields and nutrient content, potentially leading to the increased consumption of processed and energy-dense foods [105]. Additionally, air pollution has been associated with increased oxidative stress and inflammation, which may alter appetite regulation and food preferences, promoting the consumption of high-calorie and high-fat foods [106]. These changes in dietary patterns can further exacerbate the risk of obesity and metabolic disorders. Moreover, increased CO_2_ concentrations in the atmosphere result in decreased plant protein content and micronutrients, such as calcium, iron, and zinc. For example, C3 grains and tubers, such as rice, wheat, barley, and potatoes, have experienced a 7–15% decrease in protein content [107]. Thus, climate change and air pollution have a clear nutritional effect that can reduce or worsen food availability and dietary diversity.

## 5. Climate Change Adaptation and Mitigation Strategies

Climate change and air pollution are global threats that accelerate antimicrobial resistance, food and airborne diseases, and metabolic disorders. Climate change reduces crop yields and their micronutrient content, disrupting the food supply chain and increasing obesity rates [108]. Minimizing GHG emissions will help to reduce climate change impacts to a large extent [109]. The use of fossil fuels increases GHG emissions, obesity, and metabolic dysfunction. Increased physical activity, such as walking or biking, can decrease the prevalence of obesity [110]. A sustainable diet with a low microenvironmental impact is safe and could help reduce obesity and its dire consequences. Reducing meat consumption can significantly decrease GHG generation, thus indirectly impacting crop growth [111].

Incorporating plant-based proteins, such as soy, legumes, and nuts, into the diet has been suggested as a potential strategy with which to mitigate the effects of climate change and pollution-induced obesity [112]. These protein sources have a lower environmental impact compared to animal-based proteins, and their consumption has been associated with better weight management and a reduced risk of obesity-related comorbidities. Furthermore, promoting active transportation, such as walking and cycling, can contribute to a decrease in air pollution, particularly in the form of PM2.5, which has been linked to an increased risk of obesity [113].

Given the multiple threats posed by factors, such as heat, pollution, and extreme weather events, that exacerbate diabetes, the implementation of various mitigation strategies and individual adaptation measures is crucial. These strategies may include personal cooling techniques during periods of extreme heat, efforts to minimize the effects of air pollution through lifestyle modifications that reduce GHG emissions, and limiting outdoor activities or wearing face masks to minimize exposure to high levels of air pollution [114,115]. Additionally, BAT activation in cold environments has been shown to increase lipid oxidation and glucose uptake in skeletal muscle, leading to improved insulin sensitivity [32]. This highlights the potential benefits of cold exposure as a therapeutic approach to managing diabetes.

Moreover, shifting from vehicle transportation to cycling would increase physical activity levels and contribute to a reduction in GHG emissions, which might ultimately lower the risk of developing T2DM [116]. Promoting active transportation and encouraging the adoption of low-carbon transportation models could have significant health benefits while simultaneously addressing environmental concerns.

## 6. Conclusions

This review highlights the significant impact of climate change and air pollution on adipose tissue dysfunction, obesity, and metabolic health. The rising temperatures associated with global warming can impair BAT thermogenesis and adaptive energy expenditure, contributing to increased adiposity. Air pollution, particularly exposure to PM2.5, can induce WAT inflammation, oxidative stress, and mitochondrial dysfunction, exacerbating the risk of IR and metabolic disorders. Furthermore, climate change and air pollution can alter dietary patterns, promoting the consumption of energy-dense and processed foods, further contributing to the obesity epidemic.

The bidirectional relationship between obesity and climate change is evident in the current literature. The impact of climate change, particularly the increase in ambient temperatures, is expected to contribute to higher rates of obesity and T2DM, partially due to reduced physical activity levels. As the global climate continues to change, developing and implementing individual and collective strategies will be crucial for minimizing the adverse effects on public health.

Reducing the adverse effects of climate change and air pollution on metabolic health requires the implementation of policies and interventions to reduce GHG emissions, improve air quality, and promote healthy dietary habits. These may include promoting renewable energy sources, applying energy-efficient technologies, and encouraging sustainable land-use practices. Furthermore, public health initiatives that focus on promoting healthy eating habits and reducing the intake of processed foods could contribute to individual health and environmental sustainability.

## Data Availability

No new data were created or analyzed in this study. Data sharing is not applicable to this article.

## Abbreviations

ACC	Acetyl-CoA carboxylase
AHR	Aryl hydrocarbon receptor
ATGL	Adipose triglyceride lipase
BAT	Brown adipose tissue
BeAT	Beige adipose tissue
BMI	Body mass index
BPA	Bisphenol A
CFC	Chlorofluorocarbon
CREB-α	cAMP response element-binding protein alpha
CVD	Cardiovascular diseases
DDE	Dichlordiphenylethylene
DDT	Dichlorodiphenyltrichloroethane
DGAT2	Diacylglycerol O-acyltransferase 2
ER	Endoplasmic reticulum
FFA	Free fatty acid
FGF21	Fibroblast growth factor 21
GDF-15	Growth differentiation factor 15
GHG	Greenhouse gas
GLUT1	Glucose transporter type 1
GLUT4	Glucose transporter type 4
HFC	Hydrofluorocarbons
HDL	High-density lipoprotein
HOXC9	Homeobox protein C9
ICE	Intermittent cold exposure
IGFBP3	Insulin-like growth factor binding protein 3
IL-6	Interleukin-6
IR	Insulin resistance
iWAT	Inguinal WAT
LPL	Lipoprotein lipase
LYG6	Lymphocyte antigen 6 complex, locus G
MEHP	Mono-2-ethylhexyl phthalate
NF-κB	Nuclear factor-kappa B
NOx	Nitrogen oxides
NST	Non-shivering thermogenesis
PDK1	Phosphoinositide-dependent kinase-1
PGC-1α	Peroxisome proliferator-activated receptor-gamma co-activator-1-alpha
PFAS	Polyfluoroalkyl substances
PFOA	Perfluorooctanoic acid
PFOS	Perfluorooctane sulfonate
PI3K	Phosphatidylinositol 3-kinase
PKB	Protein kinase B (or Akt)
PM	Particulate matter
PM2.5	Particulate matter up to 2.5 μm
PPARα	Peroxisome proliferator-activated receptor-alpha
PPARγ	Peroxisome proliferator-activated receptor-gamma
ROS	Reactive oxygen species
sWAT	Subcutaneous WAT
T2DM	Type 2 diabetes mellitus
T3	Triiodothyronine
T4	Thyroxine
TG	Triglyceride
TN	Thermoneutral
TNFα	Tumor necrosis factor alpha
UCP1	Uncoupled protein 1
vWAT	Visceral WAT
WAT	White adipose tissue

## References

[1] Carpentier A.C., Blondin D.P., Virtanen K.A., Richard D., Haman F., Turcotte É.E. (2018). Brown Adipose Tissue Energy Metabolism in Humans. Front. Endocrinol..

[2] Jackson E., Shoemaker R., Larian N., Cassis L. (2017). Adipose Tissue as a Site of Toxin Accumulation. Comprehensive Physiology.

[3] Leitner D.R., Frühbeck G., Yumuk V., Schindler K., Micic D., Woodward E., Toplak H. (2017). Obesity and Type 2 Diabetes: Two Diseases with a Need for Combined Treatment Strategies—EASO Can Lead the Way. Obes. Facts.

[4] Klein S., Gastaldelli A., Yki-Järvinen H., Scherer P.E. (2022). Why does obesity cause diabetes?. Cell Metab..

[5] Eskenazi B., Chevrier J., Rosas L.G., Anderson H.A., Bornman M.S., Bouwman H., Chen A., Cohn B.A., de Jager C., Henshel D.S. (2009). The Pine River Statement: Human Health Consequences of DDT Use. Environ. Health Perspect..

[6] Zhang G., Sun Q., Liu C. (2016). Influencing Factors of Thermogenic Adipose Tissue Activity. Front. Physiol..

[7] Cohen P., Kajimura S. (2021). The cellular and functional complexity of thermogenic fat. Nat. Rev. Mol. Cell Biol..

[8] Luo L., Liu M. (2016). Adipose tissue in control of metabolism. J. Endocrinol..

[9] Harvey I., Boudreau A., Stephens J.M. (2020). Adipose tissue in health and disease. Open Biol..

[10] Zhang T., Chen J., Tang X., Luo Q., Xu D., Yu B. (2019). Interaction between adipocytes and high-density lipoprotein:new insights into the mechanism of obesity-induced dyslipidemia and atherosclerosis. Lipids Health Dis..

[11] Chadt A., Al-Hasani H. (2020). Glucose transporters in adipose tissue, liver, and skeletal muscle in metabolic health and disease. Pflug. Arch..

[12] Zhao S., Li N., Zhu Y., Straub L., Zhang Z., Wang M.-Y., Zhu Q., Kusminski C.M., Elmquist J.K., Scherer P.E. (2020). Partial leptin deficiency confers resistance to diet-induced obesity in mice. Mol. Metab..

[13] Fang H., Judd R.L. (2018). Adiponectin Regulation and Function. Comprehensive Physiology.

[14] de Lange P., Lombardi A., Silvestri E., Cioffi F., Giacco A., Iervolino S., Petito G., Senese R., Lanni A., Moreno M. (2023). Physiological Approaches Targeting Cellular and Mitochondrial Pathways Underlying Adipose Organ Senescence. Int. J. Mol. Sci..

[15] Goossens G.H., Blaak E.E. (2015). Adipose tissue dysfunction and impaired metabolic health in human obesity: A matter of oxygen?. Front. Endocrinol..

[16] Morigny P., Houssier M., Mouisel E., Langin D. (2016). Adipocyte lipolysis and insulin resistance. Biochimie.

[17] Chouchani E.T., Kazak L., Spiegelman B.M. (2019). New Advances in Adaptive Thermogenesis: UCP1 and Beyond. Cell Metab..

[18] Cypess A.M. (2022). Reassessing Human Adipose Tissue. N. Engl. J. Med..

[19] White U. (2023). Adipose tissue expansion in obesity, health, and disease. Front. Cell Dev. Biol..

[20] Giroud M., Jodeleit H., Prentice K.J., Bartelt A. (2022). Adipocyte function and the development of cardiometabolic disease. J. Physiol..

[21] White U.A., Fitch M.D., Beyl R.A., Hellerstein M.K., Ravussin E. (2017). Association of In Vivo Adipose Tissue Cellular Kinetics With Markers of Metabolic Health in Humans. J. Clin. Endocrinol. Metab..

[22] Longo M., Zatterale F., Naderi J., Parrillo L., Formisano P., Raciti G.A., Beguinot F., Miele C. (2019). Adipose Tissue Dysfunction as Determinant of Obesity-Associated Metabolic Complications. Int. J. Mol. Sci..

[23] Rodríguez A., Becerril S., Ezquerro S., Méndez-Giménez L., Frühbeck G. (2017). Crosstalk between adipokines and myokines in fat browning. Acta Physiol..

[24] Oka M., Kobayashi N., Matsumura K., Nishio M., Nakano K., Okamura T., Okochi H., Minamisawa T., Shiba K., Saeki K. (2020). New Role for Growth/Differentiation Factor 15 in the Survival of Transplanted Brown Adipose Tissues in Cooperation with Interleukin-6. Cells.

[25] Stanford K.I., Middelbeek R.J.W., Townsend K.L., An D., Nygaard E.B., Hitchcox K.M., Markan K.R., Nakano K., Hirshman M.F., Tseng Y.-H. (2013). Brown adipose tissue regulates glucose homeostasis and insulin sensitivity. J. Clin. Investig..

[26] Becher T., Palanisamy S., Kramer D.J., Eljalby M., Marx S.J., Wibmer A.G., Butler S.D., Jiang C.S., Vaughan R., Schöder H. (2021). Brown adipose tissue is associated with cardiometabolic health. Nat. Med..

[27] Haman F., Mantha O.L., Cheung S.S., DuCharme M.B., Taber M., Blondin D.P., McGarr G.W., Hartley G.L., Hynes Z., Basset F.A. (2016). Oxidative fuel selection and shivering thermogenesis during a 12- and 24-h cold-survival simulation. J. Appl. Physiol..

[28] Bal N.C., Maurya S.K., Pani S., Sethy C., Banerjee A., Das S., Patnaik S., Kundu C.N. (2017). Mild cold induced thermogenesis: Are BAT and skeletal muscle synergistic partners?. Biosci. Rep..

[29] Suchacki K.J., Stimson R.H. (2021). Nutritional Regulation of Human Brown Adipose Tissue. Nutrients.

[30] Cypess A.M., Weiner L.S., Roberts-Toler C., Franquet Elía E., Kessler S.H., Kahn P.A., English J., Chatman K., Trauger S.A., Doria A. (2015). Activation of human brown adipose tissue by a β3-adrenergic receptor agonist. Cell Metab..

[31] Warner A., Mittag J. (2016). Breaking BAT: Can browning create a better white?. J. Endocrinol..

[32] Symonds M.E., Farhat G., Aldiss P., Pope M., Budge H. (2019). Brown adipose tissue and glucose homeostasis—The link between climate change and the global rise in obesity and diabetes. Adipocyte.

[33] An R., Ji M., Zhang S. (2018). Global warming and obesity: A systematic review. Obes. Rev..

[34] Lim C.L. (2020). Fundamental Concepts of Human Thermoregulation and Adaptation to Heat: A Review in the Context of Global Warming. Int. J. Environ. Res. Public Health.

[35] Annesi-Maesano I. (2016). United Nations Climate Change Conferences: COP21 a lost opportunity for asthma and allergies and preparing for COP22. J. Allergy Clin. Immunol..

[36] Bastías-Pérez M., Zagmutt S., Soler-Vázquez M.C., Serra D., Mera P., Herrero L. (2020). Impact of Adaptive Thermogenesis in Mice on the Treatment of Obesity. Cells.

[37] Bal N.C., Periasamy M. (2020). Uncoupling of sarcoendoplasmic reticulum calcium ATPase pump activity by sarcolipin as the basis for muscle non-shivering thermogenesis. Philos. Trans. R. Soc. B Biol. Sci..

[38] van der Lans A.A.J.J., Hoeks J., Brans B., Vijgen G.H.E.J., Visser M.G.W., Vosselman M.J., Hansen J., Jörgensen J.A., Wu J., Mottaghy F.M. (2013). Cold acclimation recruits human brown fat and increases nonshivering thermogenesis. J. Clin. Investig..

[39] Machado S.A., Pasquarelli-do-Nascimento G., da Silva D.S., Farias G.R., de Oliveira Santos I., Baptista L.B., Magalhães K.G. (2022). Browning of the white adipose tissue regulation: New insights into nutritional and metabolic relevance in health and diseases. Nutr. Metab..

[40] de Jong J.M.A., Sun W., Pires N.D., Frontini A., Balaz M., Jespersen N.Z., Feizi A., Petrovic K., Fischer A.W., Bokhari M.H. (2019). Human brown adipose tissue is phenocopied by classical brown adipose tissue in physiologically humanized mice. Nat. Metab..

[41] Bargut T.C.L., Souza-Mello V., Aguila M.B., Mandarim-de-Lacerda C.A. (2017). Browning of white adipose tissue: Lessons from experimental models. Horm. Mol. Biol. Clin. Investig..

[42] Pirzgalska R.M., Seixas E., Seidman J.S., Link V.M., Sánchez N.M., Mahú I., Mendes R., Gres V., Kubasova N., Morris I. (2017). Sympathetic neuron–associated macrophages contribute to obesity by importing and metabolizing norepinephrine. Nat. Med..

[43] World Health Organization (2024). Obesity and Overweight. https://www.who.int/news-room/fact-sheets/detail/obesity-and-overweight.

[44] Ampofo A.G., Boateng E.B. (2020). Beyond 2020: Modelling obesity and diabetes prevalence. Diabetes Res. Clin. Pract..

[45] Tan X., Liu Y., Dong H., Xiao Y., Zhao Z. (2022). The health consequences of greenhouse gas emissions: A potential pathway. Environ. Geochem. Health.

[46] Kanazawa S. (2020). Does global warming contribute to the obesity epidemic?. Environ. Res..

[47] Magkos F., Tetens I., Bügel S.G., Felby C., Schacht S.R., Hill J.O., Ravussin E., Astrup A. (2020). The Environmental Foodprint of Obesity. Obesity.

[48] da Silva M.M., Gonçalves C.F.L., Miranda-Alves L., Fortunato R.S., Carvalho D.P., Ferreira A.C.F. (2019). Inhibition of Type 1 Iodothyronine Deiodinase by Bisphenol A. Horm. Metab. Res..

[49] Bakker L.E.H., Boon M.R., van der Linden R.A.D., Arias-Bouda L.P., van Klinken J.B., Smit F., Verberne H.J., Jukema J.W., Tamsma J.T., Havekes L.M. (2014). Brown adipose tissue volume in healthy lean south Asian adults compared with white Caucasians: A prospective, case-controlled observational study. Lancet Diabetes Endocrinol..

[50] Lidell M.E., Betz M.J., Enerbäck S. (2014). Brown adipose tissue and its therapeutic potential. J. Intern. Med..

[51] Yoneshiro T., Aita S., Matsushita M., Kayahara T., Kameya T., Kawai Y., Iwanaga T., Saito M. (2013). Recruited brown adipose tissue as an antiobesity agent in humans. J. Clin. Investig..

[52] Cypess A.M., Lehman S., Williams G., Tal I., Rodman D., Goldfine A.B., Kuo F.C., Palmer E.L., Tseng Y.-H., Doria A. (2009). Identification and importance of brown adipose tissue in adult humans. N. Engl. J. Med..

[53] van Marken Lichtenbelt W.D., Vanhommerig J.W., Smulders N.M., Drossaerts J.M.A.F.L., Kemerink G.J., Bouvy N.D., Schrauwen P., Teule G.J.J. (2009). Cold-activated brown adipose tissue in healthy men. N. Engl. J. Med..

[54] Johnson F., Mavrogianni A., Ucci M., Vidal-Puig A., Wardle J. (2011). Could increased time spent in a thermal comfort zone contribute to population increases in obesity?. Obes. Rev..

[55] Pontzer H., Durazo-Arvizu R., Dugas L.R., Plange-Rhule J., Bovet P., Forrester T.E., Lambert E.V., Cooper R.S., Schoeller D.A., Luke A. (2016). Constrained Total Energy Expenditure and Metabolic Adaptation to Physical Activity in Adult Humans. Curr. Biol..

[56] Westerterp K.R. (2004). Diet induced thermogenesis. Nutr. Metab..

[57] Donahoo W.T., Levine J.A., Melanson E.L. (2004). Variability in energy expenditure and its components. Curr. Opin. Clin. Nutr. Metab. Care.

[58] Morley N.J., Lewis J.W. (2014). Temperature stress and parasitism of endothermic hosts under climate change. Trends Parasitol..

[59] Vanhaecke T., Perrier E.T., Melander O. (2020). A Journey through the Early Evidence Linking Hydration to Metabolic Health. Ann. Nutr. Metab..

[60] Nakamura K., Velho G., Bouby N. (2017). Vasopressin and metabolic disorders: Translation from experimental models to clinical use. J. Intern. Med..

[61] Blauw L.L., Aziz N.A., Tannemaat M.R., Blauw C.A., de Craen A.J., Pijl H., Rensen P.C.N. (2017). Diabetes incidence and glucose intolerance prevalence increase with higher outdoor temperature. BMJ Open Diabetes Res. Care.

[62] Ribble A., Hellmann J., Conklin D.J., Bhatnagar A., Haberzettl P. (2023). Fine particulate matter (PM_2.5_)-induced pulmonary oxidative stress contributes to increases in glucose intolerance and insulin resistance in a mouse model of circadian dyssynchrony. Sci. Total Environ..

[63] Zhong J., Zhao G., Edwards S., Tran J., Rajagopalan S., Rao X. (2023). Particulate air pollution exaggerates diet-induced insulin resistance through NLRP3 inflammasome in mice. Environ. Pollut..

[64] Yang Z., Dong H., Gao Y., Liu S., Chen L., Ni G., Guo X., Wang M., Wang C., Chen Y. (2024). Airborne Nanoplastics Exposure Inducing Irreversible Glucose Increase and Complete Hepatic Insulin Resistance. Environ. Sci. Technol..

[65] Gong X., Wang S., Wang X., Zhong S., Yuan J., Zhong Y., Jiang Q. (2024). Long-term exposure to air pollution and risk of insulin resistance: A systematic review and meta-analysis. Ecotoxicol. Environ. Saf..

[66] Zhang K., Chen G., He J., Chen Z., Pan M., Tong J., Liu F., Xiang H. (2024). DNA methylation mediates the effects of PM_2.5_ and O_3_ on ceramide metabolism: A novel mechanistic link between air pollution and insulin resistance. J. Hazard. Mater..

[67] Pope C.A., Coleman N., Pond Z.A., Burnett R.T. (2020). Fine particulate air pollution and human mortality: 25+ years of cohort studies. Environ. Res..

[68] Della Guardia L., Shin A.C. (2022). White and brown adipose tissue functionality is impaired by fine particulate matter (PM_2.5_) exposure. J. Mol. Med..

[69] Burkart K., Causey K., Cohen A.J., Wozniak S.S., Salvi D.D., Abbafati C., Adekanmbi V., Adsuar J.C., Ahmadi K., Alahdab F. (2022). Estimates, trends, and drivers of the global burden of type 2 diabetes attributable to PM2·5 air pollution, 1990–2019: An analysis of data from the Global Burden of Disease Study 2019. Lancet Planet. Health.

[70] La Merrill M., Karey E., Moshier E., Lindtner C., La Frano M.R., Newman J.W., Buettner C. (2014). Perinatal Exposure of Mice to the Pesticide DDT Impairs Energy Expenditure and Metabolism in Adult Female Offspring. PLoS ONE.

[71] Xu C.-X., Wang C., Zhang Z.-M., Jaeger C.D., Krager S.L., Bottum K.M., Liu J., Liao D.-F., Tischkau S.A. (2015). Aryl hydrocarbon receptor deficiency protects mice from diet-induced adiposity and metabolic disorders through increased energy expenditure. Int. J. Obes..

[72] Di Gregorio I., Busiello R.A., Burgos Aceves M.A., Lepretti M., Paolella G., Lionetti L. (2019). Environmental Pollutants Effect on Brown Adipose Tissue. Front. Physiol..

[73] Feige J.N., Gerber A., Casals-Casas C., Yang Q., Winkler C., Bedu E., Bueno M., Gelman L., Auwerx J., Gonzalez F.J. (2010). The Pollutant Diethylhexyl Phthalate Regulates Hepatic Energy Metabolism via Species-Specific PPARα-Dependent Mechanisms. Environ. Health Perspect..

[74] Farrugia F., Aquilina A., Vassallo J., Pace N.P. (2021). Bisphenol A and Type 2 Diabetes Mellitus: A Review of Epidemiologic, Functional, and Early Life Factors. Int. J. Environ. Res. Public Health.

[75] Shabalina I.G., Kalinovich A.V., Cannon B., Nedergaard J. (2016). Metabolically inert perfluorinated fatty acids directly activate uncoupling protein 1 in brown-fat mitochondria. Arch. Toxicol..

[76] John L.M., Petersen N., Gerstenberg M.K., Torz L., Pedersen K., Christoffersen B.Ø., Kuhre R.E. (2022). Housing-temperature reveals energy intake counter-balances energy expenditure in normal-weight, but not diet-induced obese, male mice. Commun. Biol..

[77] Ganeshan K., Chawla A. (2017). Warming the mouse to model human diseases. Nat. Rev. Endocrinol..

[78] Kaiyala K.J., Ogimoto K., Nelson J.T., Muta K., Morton G.J. (2016). Physiological role for leptin in the control of thermal conductance. Mol. Metab..

[79] Stemmer K., Kotzbeck P., Zani F., Bauer M., Neff C., Müller T.D., Pfluger P.T., Seeley R.J., Divanovic S. (2015). Thermoneutral housing is a critical factor for immune function and diet-induced obesity in C57BL/6 nude mice. Int. J. Obes..

[80] Shankar K., Kumar D., Gupta S., Varshney S., Rajan S., Srivastava A., Gupta A., Gupta A.P., Vishwakarma A.L., Gayen J.R. (2019). Role of brown adipose tissue in modulating adipose tissue inflammation and insulin resistance in high-fat diet fed mice. Eur. J. Pharmacol..

[81] Sass F., Schlein C., Jaeckstein M.Y., Pertzborn P., Schweizer M., Schinke T., Ballabio A., Scheja L., Heeren J., Fischer A.W. (2021). TFEB deficiency attenuates mitochondrial degradation upon brown adipose tissue whitening at thermoneutrality. Mol. Metab..

[82] Spiljar M., Steinbach K., Rigo D., Suárez-Zamorano N., Wagner I., Hadadi N., Vincenti I., Page N., Klimek B., Rochat M.-A. (2021). Cold exposure protects from neuroinflammation through immunologic reprogramming. Cell Metab..

[83] Williams J.W., Elvington A., Ivanov S., Kessler S., Luehmann H., Baba O., Saunders B.T., Kim K.-W., Johnson M.W., Craft C.S. (2017). Thermoneutrality but Not UCP1 Deficiency Suppresses Monocyte Mobilization Into Blood. Circ. Res..

[84] Presby D.M., Jackman M.R., Rudolph M.C., Sherk V.D., Foright R.M., Houck J.A., Johnson G.C., Orlicky D.J., Melanson E.L., Higgins J.A. (2019). Compensation for cold-induced thermogenesis during weight loss maintenance and regain. Am. J. Physiol. Metab..

[85] Søberg S., Löfgren J., Philipsen F.E., Jensen M., Hansen A.E., Ahrens E., Nystrup K.B., Nielsen R.D., Sølling C., Wedell-Neergaard A.-S. (2021). Altered brown fat thermoregulation and enhanced cold-induced thermogenesis in young, healthy, winter-swimming men. Cell Rep. Med..

[86] González-García I., Milbank E., Diéguez C., López M., Contreras C. (2019). Glucagon, GLP-1 and Thermogenesis. Int. J. Mol. Sci..

[87] Ravussin Y., Xiao C., Gavrilova O., Reitman M.L. (2014). Effect of Intermittent Cold Exposure on Brown Fat Activation, Obesity, and Energy Homeostasis in Mice. PLoS ONE.

[88] Boström P., Wu J., Jedrychowski M.P., Korde A., Ye L., Lo J.C., Rasbach K.A., Boström E.A., Choi J.H., Long J.Z. (2012). A PGC1-α-dependent myokine that drives brown-fat-like development of white fat and thermogenesis. Nature.

[89] Weng X., Wang C., Yuan Y., Wang Z., Kuang J., Yan X., Chen H. (2023). Effect of Cold Exposure and Exercise on Insulin Sensitivity and Serum Free Fatty Acids in Obese Rats. Med. Sci. Sport. Exerc..

[90] Mancini S.J., White A.D., Bijland S., Rutherford C., Graham D., Richter E.A., Viollet B., Touyz R.M., Palmer T.M., Salt I.P. (2017). Activation of AMP-activated protein kinase rapidly suppresses multiple pro-inflammatory pathways in adipocytes including IL-1 receptor-associated kinase-4 phosphorylation. Mol. Cell. Endocrinol..

[91] Xu R., Zhong Y., Li R., Li Y., Zhong Z., Liu T., Wang Q., Lv Z., Huang S., Duan Y.-G. (2023). Association between exposure to ambient air pollution and semen quality: A systematic review and meta-analysis. Sci. Total Environ..

[92] Mendez R., Zheng Z., Fan Z., Rajagopalan S., Sun Q., Zhang K. (2013). Exposure to fine airborne particulate matter induces macrophage infiltration, unfolded protein response, and lipid deposition in white adipose tissue. Am. J. Transl. Res..

[93] Wang N., Ma Y., Liu Z., Liu L., Yang K., Wei Y., Liu Y., Chen X., Sun X., Wen D. (2019). Hydroxytyrosol prevents PM_2.5_-induced adiposity and insulin resistance by restraining oxidative stress related NF-κB pathway and modulation of gut microbiota in a murine model. Free Radic. Biol. Med..

[94] Campolim C.M., Weissmann L., Ferreira C.K.D.O., Zordão O.P., Dornellas A.P.S., de Castro G., Zanotto T.M., Boico V.F., Quaresma P.G.F., Lima R.P.A. (2020). Short-term exposure to air pollution (PM_2.5_) induces hypothalamic inflammation, and long-term leads to leptin resistance and obesity via Tlr4/Ikbke in mice. Sci. Rep..

[95] Hersoug L.-G., Møller P., Loft S. (2018). Role of microbiota-derived lipopolysaccharide in adipose tissue inflammation, adipocyte size and pyroptosis during obesity. Nutr. Res. Rev..

[96] Daiber A., Kuntic M., Hahad O., Delogu L.G., Rohrbach S., Di Lisa F., Schulz R., Münzel T. (2020). Effects of air pollution particles (ultrafine and fine particulate matter) on mitochondrial function and oxidative stress—Implications for cardiovascular and neurodegenerative diseases. Arch. Biochem. Biophys..

[97] Burgoine T., Monsivais P., Sharp S.J., Forouhi N.G., Wareham N.J. (2021). Independent and combined associations between fast-food outlet exposure and genetic risk for obesity: A population-based, cross-sectional study in the UK. BMC Med..

[98] Zhao Z., Zhen S., Yan Y., Liu N., Ding D., Kong J. (2023). Association of dietary patterns with general and central obesity among Chinese adults: A longitudinal population-based study. BMC Public Health.

[99] Miguel E.D.S., Lopes S.O., Araújo S.P., Priore S.E., Alfenas R.d.C.G., Hermsdorff H.H.M. (2020). Association between food insecurity and cardiometabolic risk in adults and the elderly: A systematic review. J. Glob. Health.

[100] Nkambule S.J., Moodley I., Kuupiel D., Mashamba-Thompson T.P. (2021). Association between food insecurity and key metabolic risk factors for diet-sensitive non-communicable diseases in sub-Saharan Africa: A systematic review and meta-analysis. Sci. Rep..

[101] Turner C., Kalamatianou S., Drewnowski A., Kulkarni B., Kinra S., Kadiyala S. (2020). Food Environment Research in Low- and Middle-Income Countries: A Systematic Scoping Review. Adv. Nutr..

[102] von Braun J. (2018). Economic and Political Innovation for Nutritional Improvement. World Rev. Nutr. Diet..

[103] Owino V., Kumwenda C., Ekesa B., Parker M.E., Ewoldt L., Roos N., Lee W.T., Tome D. (2022). The impact of climate change on food systems, diet quality, nutrition, and health outcomes: A narrative review. Front. Clim..

[104] Hallström E., Bajzelj B., Håkansson N., Sjons J., Åkesson A., Wolk A., Sonesson U. (2021). Dietary climate impact: Contribution of foods and dietary patterns by gender and age in a Swedish population. J. Clean. Prod..

[105] Domingo N.G.G., Balasubramanian S., Thakrar S.K., Clark M.A., Adams P.J., Marshall J.D., Muller N.Z., Pandis S.N., Polasky S., Robinson A.L. (2021). Air quality–related health damages of food. Proc. Natl. Acad. Sci. USA.

[106] Sundram T.K.M., Tan E.S.S., Lim H.S., Amini F., Bustami N.A., Tan P.Y., Rehman N., Ho Y.B., Tan C.K. (2022). Effects of Ambient Particulate Matter (PM_2.5_) Exposure on Calorie Intake and Appetite of Outdoor Workers. Nutrients.

[107] Myers S.S., Zanobetti A., Kloog I., Huybers P., Leakey A.D.B., Bloom A.J., Carlisle E., Dietterich L.H., Fitzgerald G., Hasegawa T. (2014). Increasing CO2 threatens human nutrition. Nature.

[108] Rezaei E.E., Webber H., Asseng S., Boote K., Durand J.L., Ewert F., Martre P., MacCarthy D.S. (2023). Climate change impacts on crop yields. Nat. Rev. Earth Environ..

[109] Young B., Birney C., Ingwersen W.W. (2024). Dataset of 2012-2020 U.S. National- and State-Level Greenhouse Gas Emissions by Sector. Data Br..

[110] Flint E., Webb E., Cummins S. (2016). Change in commute mode and body-mass index: Prospective, longitudinal evidence from UK Biobank. Lancet Public Health.

[111] Haines A., Ebi K. (2019). The Imperative for Climate Action to Protect Health. N. Engl. J. Med..

[112] Hirvonen K., Bai Y., Headey D., Masters W.A. (2020). Affordability of the EAT–Lancet reference diet: A global analysis. Lancet Glob. Health.

[113] Landrigan P.J., Fuller R., Acosta N.J.R., Adeyi O., Arnold R., Basu N., Baldé A.B., Bertollini R., Bose-O’Reilly S., Boufford J.I. (2018). The Lancet Commission on pollution and health. Lancet.

[114] Jay O., Capon A., Berry P., Broderick C., de Dear R., Havenith G., Honda Y., Kovats R.S., Ma W., Malik A. (2021). Reducing the health effects of hot weather and heat extremes: From personal cooling strategies to green cities. Lancet.

[115] Carlsten C., Salvi S., Wong G.W.K., Chung K.F. (2020). Personal strategies to minimise effects of air pollution on respiratory health: Advice for providers, patients and the public. Eur. Respir. J..

[116] Dain K., Hadley L. (2012). Diabetes and climate change—Two interconnected global challenges. Diabetes Res. Clin. Pract..

